# Cutaneous Microthrombosis in a Patient With Factor V Leiden Heterozygosity and Antibodies to Phosphatidylserine/Prothrombin Complex

**DOI:** 10.7759/cureus.75977

**Published:** 2024-12-18

**Authors:** Francis A Nardella

**Affiliations:** 1 Rheumatology, St. Luke’s Meridian Medical Center, Meridian, USA

**Keywords:** antiphosphatidylserine/prothrombin complex antibodies, cutaneous microthrombosis, heterozygous factor v leiden mutation, noncriteria antiphospholipid antibodies, "seronegative" antiphospholipid antibody syndrome

## Abstract

This report describes the development of recurrent cutaneous microthrombosis in a patient with the superposition of Factor V Leiden heterozygosity on a noncriteria IgM antibody to phosphatidylserine/prothrombin complex. The patient was treated with prednisone, apixaban, and rituximab and was stable off of prednisone at her last outpatient visit 22 months after the initial event. This report illustrates the challenges of dealing with multifactor thrombophilia especially when one of those factors is a noncriteria antiphospholipid antibody and reaffirms the value of testing for noncriteria antibodies when clinical findings suggest the presence of antiphospholipid antibodies but the criteria antibodies are negative. This report further shows, in this patient, the benefit of the addition of rituximab-pvv to apixaban in normalizing the level of antiphosphatidylserine/prothrombin complex antibodies with the cessation of cutaneous microthrombotic events, normalization of inflammatory markers, and allowing the discontinuation of prednisone. Because of the relatively high frequency of Factor V Leiden heterozygosity in Caucasian populations, this report suggests that dual-factor thromobophilia due to its combination with criteria or noncriteria antiphospholipid antibodies may be more common than is recognized.

## Introduction

Cutaneous microthrombosis is one of the manifestations and one of the clinical criteria for the diagnosis of antiphospholipid antibody syndrome (APS) [1]. Factor V Leiden heterozygosity is one of the most common heritable thrombophilias that predispose to thromboembolic disease [2,3]. The risk of thromboembolism is increased when heterozygous Factor V Leiden is combined with other heritable thrombophilias, with antiphospholipid antibodies, with clinical factors such as obstetric settings and surgery, and with drugs such as oral contraceptives [2] and likely with other factors that increase the risk for thromboembolism such as infections [3]. The laboratory criteria for the diagnosis of APS are the presence of moderate to high levels of IgG and/or IgM antibodies to cardiolipin, IgG and/or IgM antibodies to B2 glycoprotein I, and/or the presence of a lupus anticoagulant [1]. Antibodies to phosphatidylserine/prothrombin complex are one of the noncriteria antiphospholipid antibodies described in “seronegative” APS [4,5]. A case is presented describing the course of a patient with Factor V Leiden heterozygosity combined with the noncriteria IgM antibody to phosphatidylserine/prothrombin complex resulting in recurrent cutaneous microthrombosis and skin necrosis.

## Case presentation

**Figure 1:**
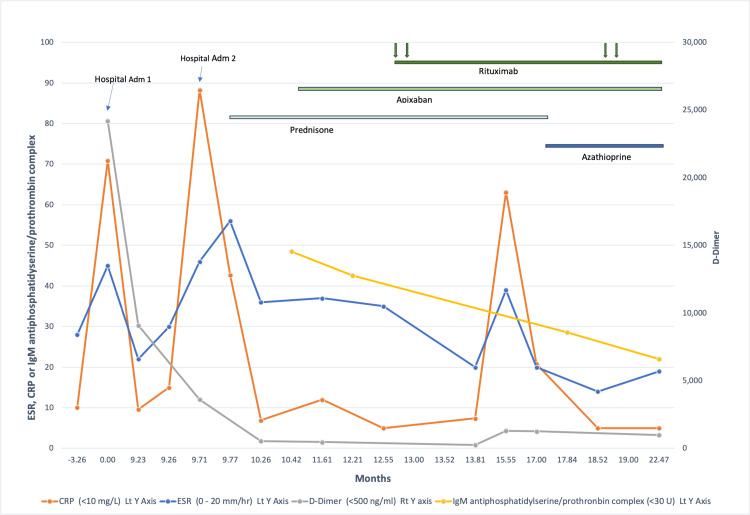
Time course of ESR, CRP, D-dimer, and antiphosphatidylserine/prothrombin complex antibody levels with reference ranges and drug therapy ESR: erythrocyte sedimentation rate; CRP: C-reactive protein

A 30-year-old woman was admitted to the hospital with purpura involving the left shoulder, abdomen, and back that had developed over one day (Time 0 in Figure 1). She had been seen in the emergency department one day earlier with a two-day history of sore throat and was treated for streptococcal pharyngitis with amoxicillin. She had a history of superficial thrombophlebitis involving the left arm seven months earlier treated with a short course of apixaban. She also had a history of inflammatory bowel disease but was not on specific therapy.

**Figure 2:**
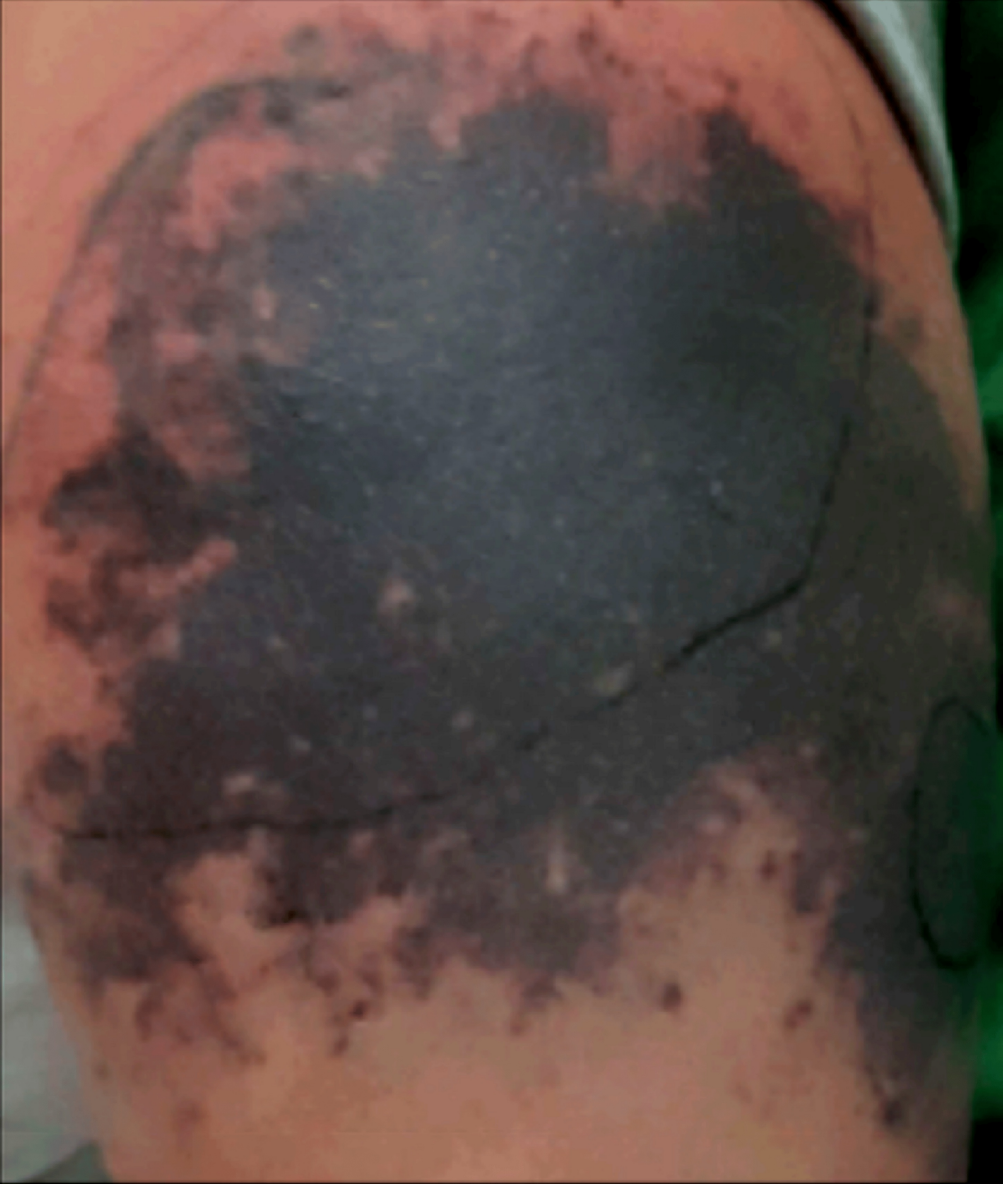
Large coalescent area of palpable purpura on the left shoulder during the first hospital admission

On physical examination, there was a large coalescent area of palpable purpura on the left shoulder (Figure 2) and a few smaller areas of purpura on the back and abdomen. The oropharynx was clear. 

She was found to be heterozygous for Factor V Leiden mutation 506Q and negative for Prothrombin G20210A mutation. IgG and IgM antibodies to cardiolipin and B2 glycoprotein I were negative, and a lupus anticoagulant was negative (see laboratory studies in Table 1). A rapid test for oral group A streptococcus was positive, and one out of two blood cultures was positive for non-group A *Streptococcus anginosus *from the aerobic culture and *Actinomyces oris* from the anaerobic culture. A complete blood count revealed a white blood count (WBC) of 21.7 (3.8-11 x 1000/ul) with 16.0 neutrophils (1.9-8 x 1000/ul) and 2.1 monocytes (0.10-0.80 x 1000/ul). Red blood cell (RBC) count, RBC indices, hemoglobin, and hematocrit were normal. Platelet count was elevated at 460 (150-420 x 1000/ul). A chemistry panel showed normal renal and normal liver function studies although a review of her record revealed fluctuating low-level elevations of alkaline phosphatase over the preceding seven years and fluctuating low-level elevations of alanine aminotransferase (ALT) over the preceding nine years. A urinalysis showed trace blood, 1+ protein with 10-25 squamous epithelial cells/high-power field (hpf) (0-5/hpf), 25-50 WBC (0-5/hpf), and 3-5 RBC/hpf (0-3/hpf). Urine culture revealed no growth.

**Table 1:** Laboratory studies including reference ranges and results ND: not detected; WNL: within normal limits; ANCA: antineutrophil cytoplasmic antibodies; ANA: antinuclear antibody

Month 0		Months 9-10		Month 20	
ANA (negative)	Negative	ANA (negative)	Negative	IgM antiphosphatidylserine antibodies (0-15 GPL)	0
Total hemolytic complement (38.7-89.9 U/ml)	89.6	Total hemolytic complement (42-95 U/ml)	70	IgG antiphosphatidylserine antibodies (0-21 MPL)	0
Lupus anticoagulant (ND)	ND	C3 compliment (80-175 mg/dl)	123	Antithrombin III (84%-124%)	124
IgG anticardiolipin antibody (<15 GPL)	<9.4	C4 compliment 12-55 mg/dl)	27		
IgM anticardiolipin antibody (<12.5 MPL)	<9.4	Protein C activity (70%-140%)	136		
IgG antiB2 glycoprotein I antibody (<20 SGL)	<10	Protein S activity (55%-146%)	116		
IgM anti B2 Glycoprotein I antibody (<20 SMU)	<10	Rheumatoid factor (<12 IU/ml)	<9		
IgA antiB2 glycoprotein I antibody (<20 SAU)	<10	Serum protein electrophoresis (WNL)	WNL		
Factor V Leiden 506Q mutatiuon (ND)	Detected	Cryooglobulin (negative)	Negative		
Prothrombin gene 20210A mutation (ND)	ND	IgG (700-1600 mg/dl)	1270		
ANCA titer (<1:20); pattern (ND)	ND	IgA (70-400 mg/dl)	322		
Antimyeloperoxidase antibodies (0-19 AU)	0	IgM (42-230 mg/dl)	161		
Antiserineproteinase 3 antibodies (0-19 AU)	95	IgG1 (382-929 mg/dl)	698		
Fibrinogen (164-424 mg/dl)	413	IgG2 (242-700 mg/dl)	458		
		IgG3 (22-176 mg/dl)	59		
		IgG4 (4-86 mg/dl)	1		
		ANCA titer (<1:20); pattern (ND)	1:40 p-ANCA		
		Antimyeloperoxidase antibody (0-19 AU/ml)	0		
		Antiserineproteinase 3 antibody (0-19 AU/ml)	243		
		Angiotensin-converting enzyme (16-85 AU/ml)	15		
		Hepatitis C antibody (ND)	ND		
		Hepatitis B surface antigen (NR)	NR		
		Hepatitis B core IgM antibody (NR)	NR		
		HIV 1 & 2, p24 Ag (ND)	ND		
		MPL codon 515 mutation (ND)	ND		
		Jak 2 (V617F) mutation (ND)	ND		
		Calreticulin exon 9 mutation (ND)	ND		

**Figure 3:**
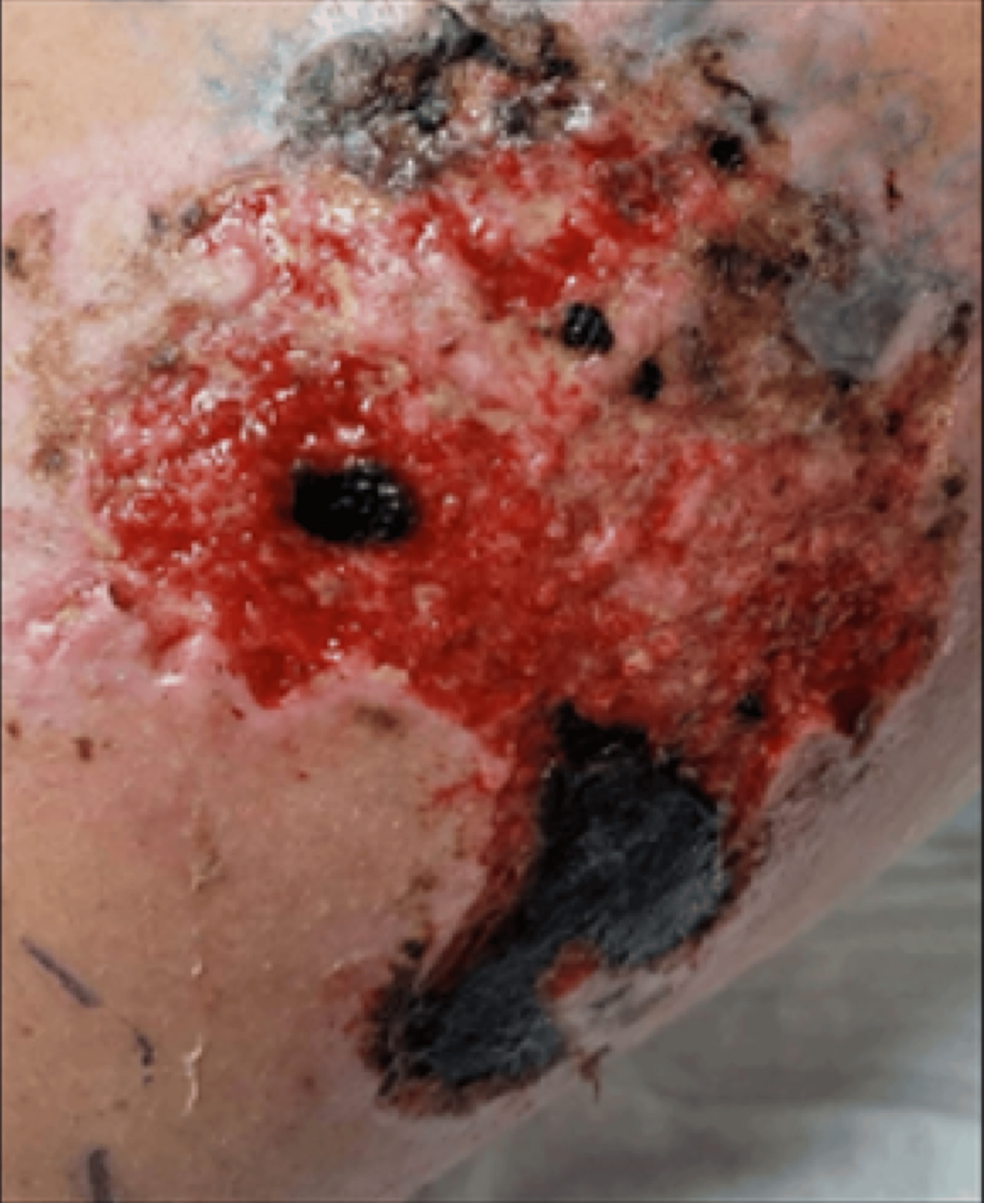
Progression of purpuric area on the skin of the left shoulder to necrosis over a period of nine days

During her two-day hospital stay, the area of purpura on the left shoulder became more pronounced. She was discharged with a 10-day course of amoxicillin. Over the course of one week following discharge, the area of purpura on the left shoulder becomes necrotic (Figure 3). The areas of purpura on the back and abdomen resolved without issue, and the lesion on the left shoulder eventually healed seven months later with residual scarring.

**Figure 4:**
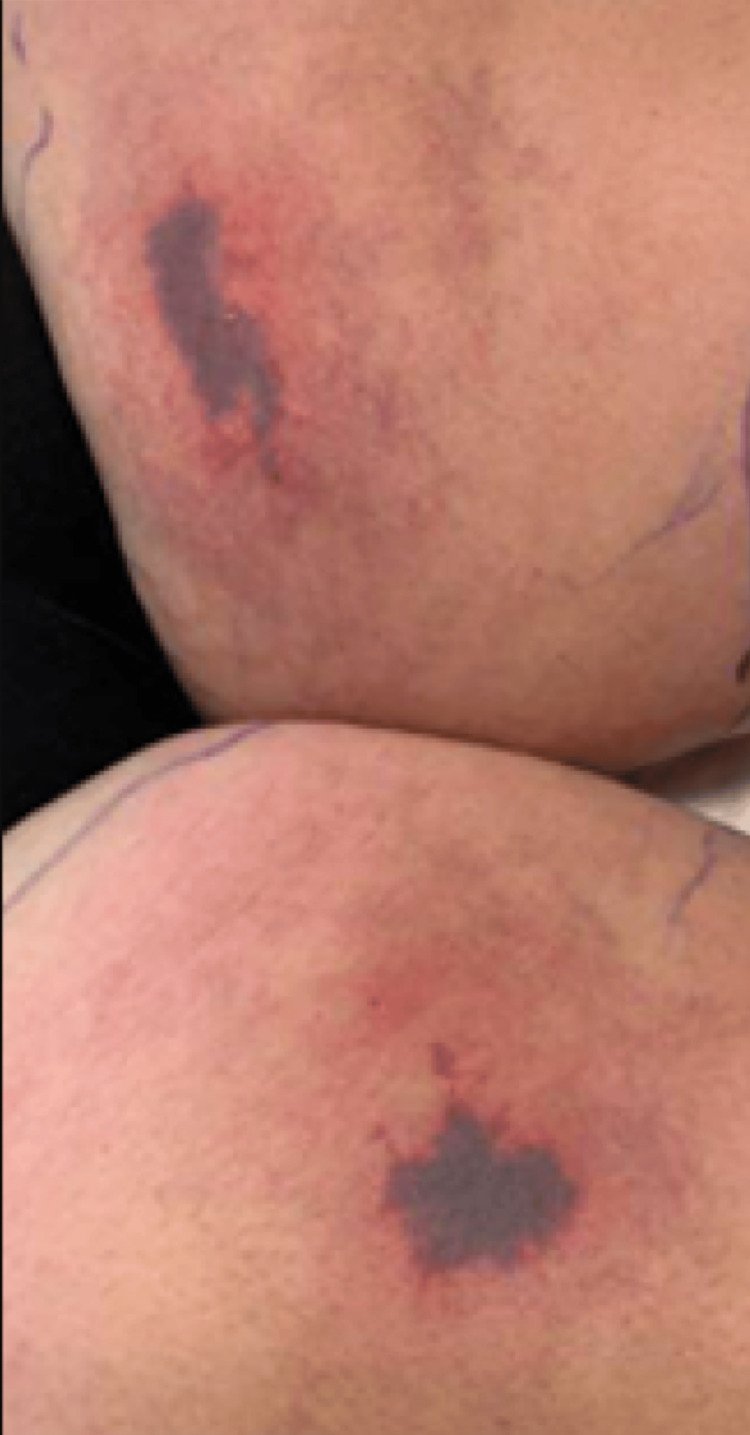
Purpuric lesions over the proximal anterior thighs at the time of the second hospital admission

At 9.7 months after the initial episode, she developed purpuric lesions over the proximal anterior thighs (Figure 4) that became necrotic. Rheumatoid factor, cryoglobulin, serum protein electrophoresis, ANA, CH50, C3, C4, quantitative IgG, IgA, IgM, and IgG subclasses were negative or normal except for the reduction in IgG4. Protein S and protein C activities were normal. Antithrombin III was tested later (month 20) and was normal. ANCA screen was positive in an atypical “cross-positive manner” with p-ANCA pattern positive at a titer of 1:40 with positive antiserineproteinase 3 antibodies at 243 U (see laboratory studies in Table 1). WBC was elevated at 15.45 with 10.02 neutrophils and 1.36 monocytes. The platelet count was elevated at 556. A review of her record showed that she had had fluctuating elevations of WBC over the preceding 11 years and fluctuating elevations of platelet count for the preceding 10 years. MPL codon 515, Jak2 V617F, and calreticulin exon 9 mutations performed at month 9 were not detected. A chemistry panel showed normal renal function with an alkaline phosphatase of 154 and an ALT of 37. Urinalysis showed trace leukocytes, negative protein, 5-10 WBC/hpf, and 0-2 RBC/hpf. Urine culture was negative.

**Figure 5:**
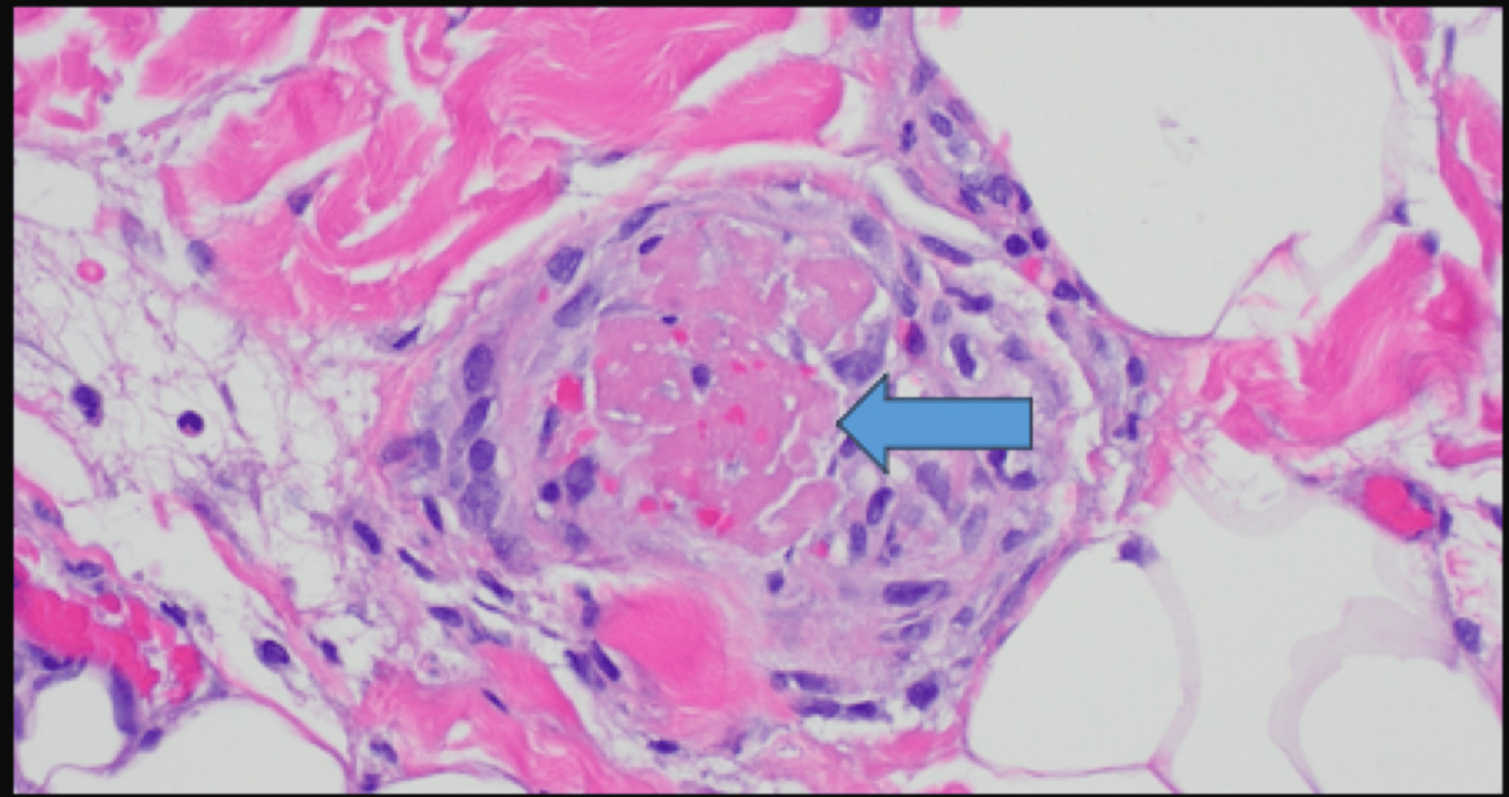
Photomicrograph of H&E stained fixed tissue obtained from biopsy of purpuric skin lesion on right thigh H&E: hematoxylin and eosin staining The arrow points to fibrin deposition in a dermal blood vessel without evidence for vasculitis

Prednisone was started because of suspected vasculitis. However, hematoxylin and eosin (H&E) staining of tissue biopsy taken from the right thigh lesion processed at the University of Utah Dermatopathology Laboratory revealed microthrombosis without vasculitis (Figure 5). 

**Figure 6:**
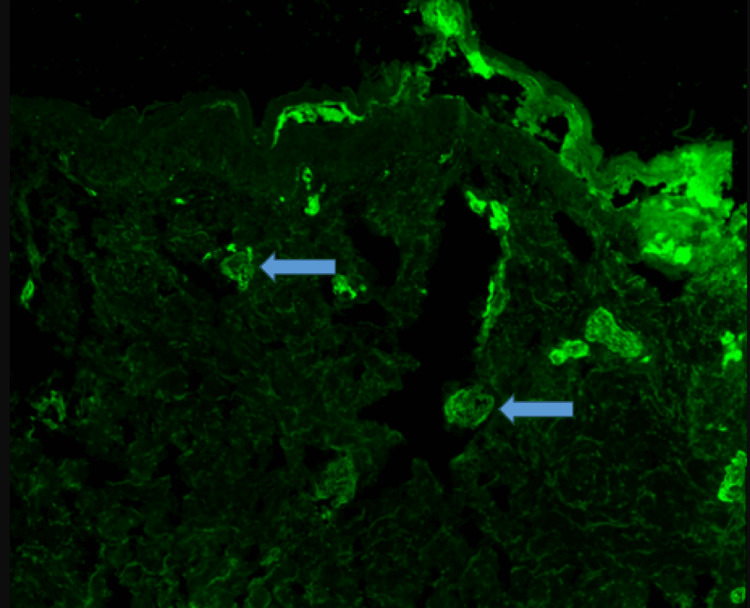
Photomicrograph of immunofluorescent staining of tissue obtained from the biopsy of a skin lesion on the proximal anterior right thigh for the presence of IgM The arrows point to areas of fluorescence indicating the presence of IgM deposition in dermal blood vessels

Immunofluorescence microscopy performed at the University of Utah Immunodermatopathology Laboratory showed staining for IgM in the superficial, upper dermal, and mid-dermal blood vessels (Figure 6) as well as for C3 and fibrinogen surrounding superficial epidermal vessels (not shown).

**Figure 7:**
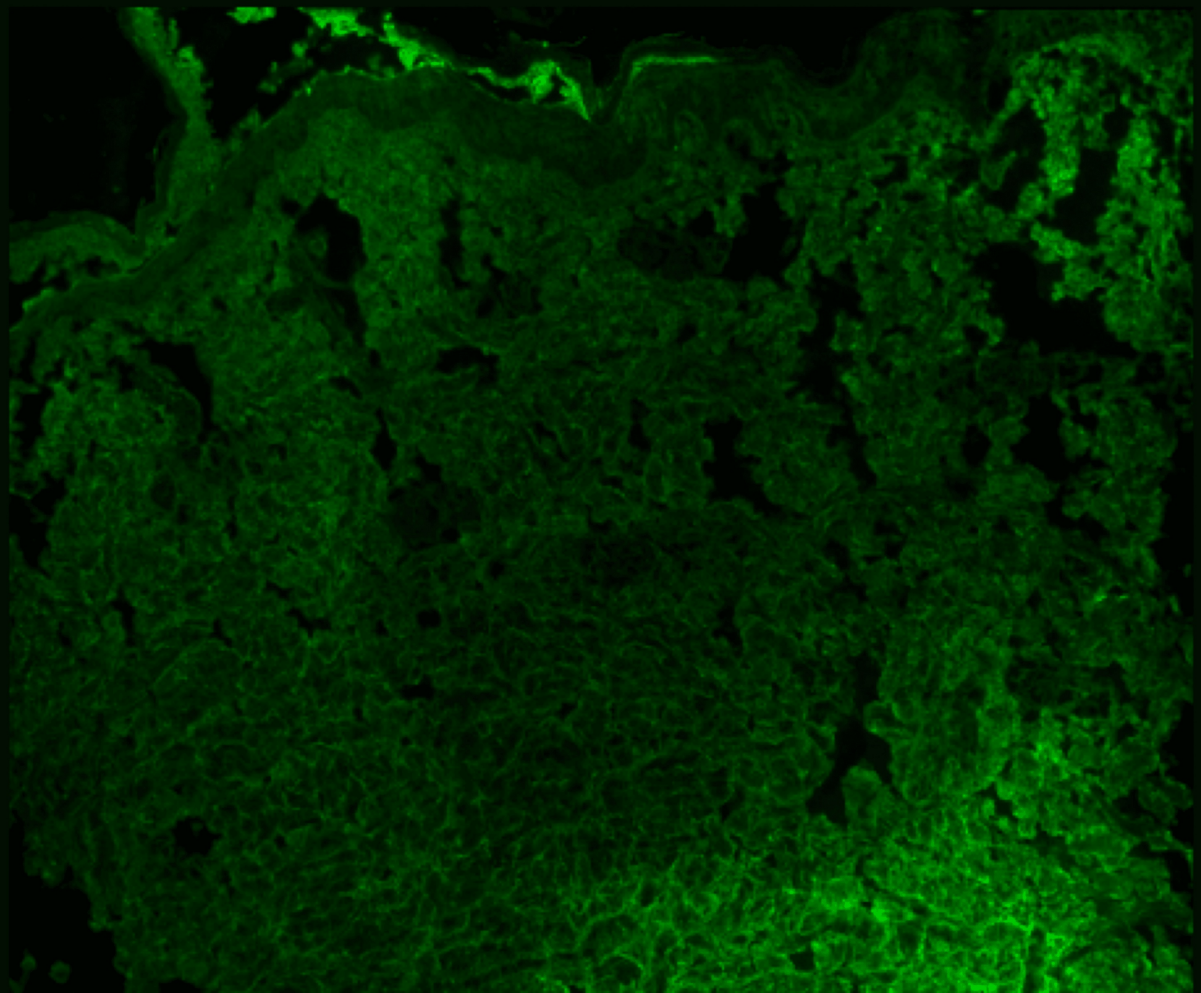
Photomicrograph of immunofluorescent staining of tissue obtained from skin biopsy from the right thigh for IgG No immunofluorescence staining is present to indicate the presence of IgG in the skin biopsy specimen

Stains for IgG including IgG4 and IgA were negative. Staining for IgG is shown in Figure 7. Stains for IgG4 and IgA are not shown.

Because of the findings on biopsy, a search for noncriteria antiphospholipid antibodies was done. IgM but not IgG antibodies to phosphatidylserine/prothrombin complex (Mayo Laboratories performed the first two assay data points and ARUP Laboratories performed the 3rd and last) was positive (see Figure 1 for the time course of antiphosphatidyserine/prothrombin complex antibody levels). IgG and IgM antibodies to phosphatidylserine done at month 20 were negative. A search for other noncriteria antibodies such as those mentioned by Devreese [4] and Truglia et al. [5] was not done. 

Apixaban was added to her regimen at month 10.4. Because of recurrent areas of painful libido with attempts at corticosteroid reduction, rituximab-pvv infusions were started at month 13 (see Figure 1 for the time course of therapies). On rituximab-pvv, the levels of antiphosphatidylserine/prothrombin complex antibodies returned to normal by month 17 (Figure 1). 

At her last outpatient visit (month 21), 10 months after the second event, she was stable off of prednisone with previous lesions healed and no new purpuric lesions on apixaban and rituximab although she had developed a second bout of superficial thrombophlebitis involving the right lower extremity at month 17. Treatment for inflammatory bowel disease was initiated at month 17 with azathioprine (Figure 1).

## Discussion

The presence of Factor V Leiden heterozygosity increases the risk for thromboembolic disease. The superposition of an additional factor further increases the risk. The negative tests for criteria antiphospholipid antibodies and lupus anticoagulant were confounding factors in her care. The pseudovasculitic nature of the process with cutaneous thrombosis and elevated inflammatory markers, as well as the negative studies for criteria antiphospholipid antibodies, was an additional confounding factor that led to the delay in diagnosis and treatment. 

The absence of vasculitis on biopsy, the presence of microvascular thrombosis, and the presence of immunofluorescent staining for IgM involving the dermal blood vessels in the absence of rheumatoid factor, cryoglobulin, and a paraprotein suggested the presence of a noncriteria antiphospholipid antibody as a cofactor in the thrombotic process. The finding of IgM antiphosphatidylserine/prothrombin complex antibodies brought clarity to the nature of the process. IgG antibodies to phosphatidylserine/prothrombin complex and IgG and IgM antibodies to phosphatidylserine were negative. A search for other noncriteria antiphospholipid antibodies was not done. The multifactor thrombophilia especially when one of the factors was a noncriteria antiphospholipid antibody was the major confounding factor in her care.

The relatively high prevalence of Factor V Leiden heterogenicity in Caucasian populations [2,3] suggests that its combination with criteria and noncriteria antiphospholipid antibodies may be more common than is recognized. The atypical “cross-positive” ANCA, p-ANCA pattern with antibodies to serine proteinase 3, was felt consistent with inflammatory bowel disease [6,7]. 

Although not standard therapy, because of the severity of the cutaneous microthrombotic disease and recurrent areas of painful erythema with attempts at corticosteroid reduction while on apixaban, rituximab-pvv was added to her regimen leading to normalization of the test for antibodies to phosphatidylserine/prothrombin complex and cessation of cutaneous thrombotic events although low-level elevations of platelets and D-dimer have persisted.

## Conclusions

This report illustrates the diagnostic and therapeutic challenges of recurrent cutaneous microthrombosis as a result of multifactor thrombophilia due to a combination of a noncriteria antiphospholipid antibody superimposed on Factor V Leiden heterozygosity. It also demonstrates the value of testing for noncriteria antiphospholipid antibodies when clinical findings suggest the presence of antiphospholipid antibodies but the criteria antibodies are negative. This report further shows, in this patient, the benefit of the addition of rituximab-pvv to apixaban in normalizing the level of antiphosphatidylserine/prothrombin complex antibodies with cessation of cutaneous microthrombotic events, normalization of inflammatory markers, and allowing the discontinuation of prednisone. Because of the relatively high frequency of Factor V Leiden heterozygosity in Caucasian populations, this report suggests that dual-factor thrombophilia due to its combination with criteria or noncriteria antiphospholipid antibodies may be more common than is recognized.
